# Brentuximab Vedotin Treatment for Primary Refractory Hodgkin Lymphoma

**DOI:** 10.1155/2013/351292

**Published:** 2013-10-02

**Authors:** Hung-Bo Wu, Shyh-An Yeh, Huei-Yung Chen

**Affiliations:** ^1^Division of Hematology and Oncology, Department of Medicine, Edah Hospital, No. 1, Yi-Da Road, Yen-Tsao District, P.O. Box 824, Kaohsiung, Taiwan; ^2^Department of Radiation Oncology, Edah Hospital, Kaohsiung, Taiwan; ^3^Department of Nuclear Medicine, Edah Hospital, Kaohsiung, Taiwan

## Abstract

Up to 40% of patients with advanced Hodgkin lymphoma (HL) become refractory or relapsed after current standard chemotherapy, among which primary refractory HL confers a particularly poor outcome. With intensive salvage chemotherapy and autologous stem cell transplantation, the long-term remission rate for these patients was only 30%, but more selective treatments with higher therapeutic index are needed. We report the experience of using a new anti-CD30 immunotoxin, brentuximab vedotin, in salvage treatment of a 30-year-old woman with primary refractory Hodgkin lymphoma. The patient presented with SVC syndrome due to the bulky mediastinal tumor and was confirmed to have classical Hodgkin lymphoma, nodular sclerosis type, stage IIIA. The tumor responded to induction chemotherapy transiently, but local progression was noted during subsequent cycles of treatment. Salvage radiotherapy to the mediastinal tumor, obtained no remission but was followed by rapid in-field progression and then lung metastasis. She declined stem cell transplantation and received salvage brentuximab vedotin (BV) therapy, which induced dramatic shrinkage of tumor without significant side effects. Serial followup of PET/CT imaging confirmed a rapid and continuous complete remission for 12 months. Although durability of the remission needs further observation, this case illustrates the excellent efficacy of brentuximab vedotin in primary refractory Hodgkin lymphoma.

## 1. Background 

Despite recent improvement of therapy, the outcome of patients with refractory or relapsed Hodgkin lymphoma (RR-HL) remained compromised [1]. In order to improve the efficacy of induction treatment, the German Hodgkin Study Group (GHSG) developed a more intensive regimen (escalated BEACOPP), which achieved higher response rate (RR) and progression-free survival (PFS), but the overall survival (OS) was not improved due to increase of toxicities [2]. Salvage chemotherapy followed by autologous stem cell transplantation (ASCT) confers a long-term survival rate of 50% for patients with relapsed HL [3]. Unfortunately, this strategy had been less successful for the primary refractory HL, with poor long-term survival (only 30%) but significant morbidities [4]. The optimal therapy for primary refractory HL remains undefined. Brentuximab vedotin (BV), an immunotoxin targeting cell-surface CD30 protein, had demonstrated efficacy in HL and was approved by FDA for treatment of HL relapse after ASCT or failure of two multiagent regimens and not candidates for ASCT. However, its role in the highly resistant primary refractory HL needs to be explored. Herein, we report a woman of primary refractory HL resistant to induction chemotherapy and salvage radiotherapy who achieved a rapid and persistent remission after BV treatment.

## 2. Case Report 

**Figure 1:**
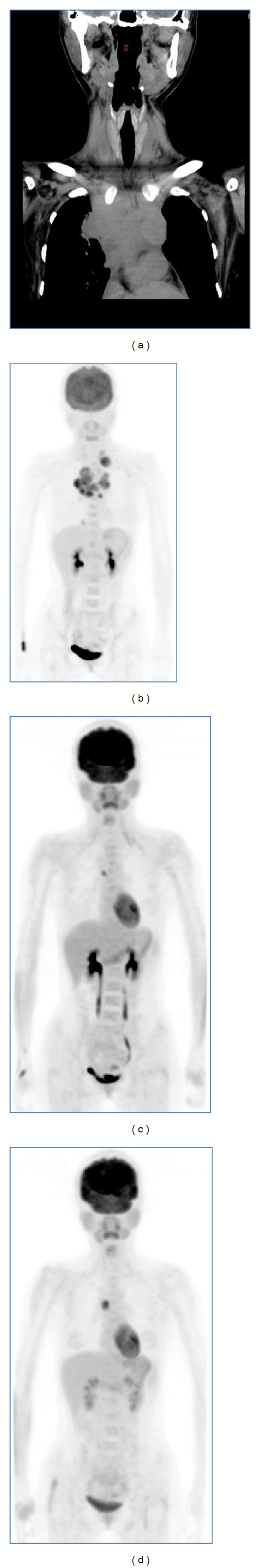
Images showing Hodgkin lymphoma refractory to induction chemotherapy. (a) Chest computerized tomography (CT) demonstrates the superior mediastinal tumor at diagnosis. (b) Positron emission tomography/computerized tomography (PET/CT) reveals involvement of right supraclavicular, left lower neck, mediastinal, epicardial, and retroperitoneal lymph nodes at initial diagnosis. (c) PET/CT after 4 cycles of induction chemotherapy shows partial remission with a residual uptake at right anterior mediastinum. (d) PET/CT after completion of 6 cycles of chemotherapy reveals relapse of the mediastinal tumor.

A thirty-year-old female patient presented with dyspnea due to SVC syndrome. Chest CT scan showed bulky tumors over the mediastinum and left supraclavicular fossa, with right internal jugular vein thrombosis and right side pleural effusion (Figure 1(a)). Biopsy of the left neck tumor confirmed classical Hodgkin lymphoma, nodular sclerosing type. PET/CT study revealed involvement of right supraclavicular, left lower neck, mediastinal, epicardial, and retroperitoneal lymph nodes (Figure 1(b)).

Under diagnosis of classical Hodgkin lymphoma, stage IIIA, she received the standard induction chemotherapy with epirubicin, bleomycin, vinblastine, and dacarbazine (EBVD), with regression of neck tumors and SVC syndrome. After four cycles of chemotherapy, an interim PET/CT study showed partial remission with residual uptake over right anterior mediastinum (Figure 1(c)). The patient declined further histological study of the mediastinal mass and continued with the planned chemotherapy. After completion of six cycles of chemotherapy, local relapse of the mediastinal tumor was found in the posttreatment PET/CT study (Figure 1(d)). 

**Figure 2:**
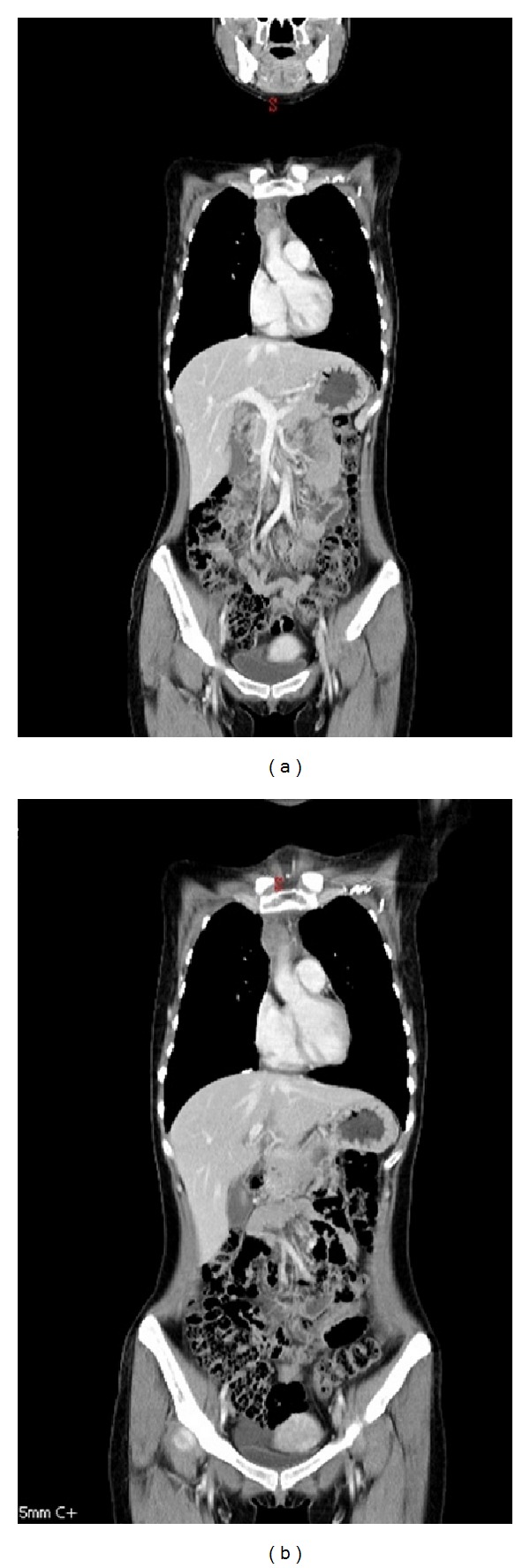
Mediastinal tumor refractory to salvage radiotherapy. (a) CT shows relapsed superior mediastinal tumor before radiotherapy. (b) CT reveals progression of the superior mediastinal tumor 2 months after completion of radiotherapy.

Salvage radiotherapy was commenced for the progressive mediastinal tumor. The PET/CT scan images and CT-simulation images were fused for contouring target volumes. The involved lymphoid regions constituted the gross tumor volume. Isodose curves confirmed that at least 95% of the prescribed dose was delivered to the planning target volume. A total of 36 Gy in 20 daily fractions, 5 fractions per week, was delivered with intensity-modulated radiotherapy techniques. The patient tolerated it well, but CT imaging showed no remission of the mediastinal tumor followed by early relapse (Figures 2(a) and 2(b)).

**Figure 3:**
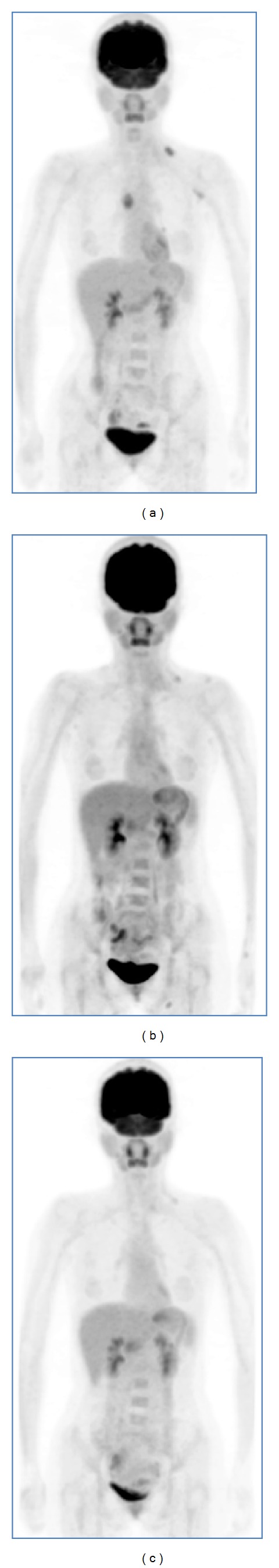
Serial PET/CT images before and after brentuximab vedotin treatment. (a) Before treatment, showing mediastinal tumor progression, with metastases to left lung, left axillary, and supraclavicular lymph nodes. (b) Near-complete remission after 4 cycles of treatment, with a faint uptake over left lower neck. (c) Complete remission after 9 cycles of treatment.

Due to immediate relapse after induction chemotherapy and no response to salvage radiotherapy, primary refractory HL was impressed. Salvage chemotherapy and ASCT was suggested, but the patient hesitated due to concerns about anticipated toxicities with high-dose chemotherapy and ASCT. Two months later, left neck tumor enlarged significantly, the PET/CT study revealed progressive mediastinal tumor with metastases to left lung, left axilla, and supraclavicular lymph nodes (Figure 3(a)). Biopsy of the left neck mass confirmed a recurrence of HL. 

Recognizing the efficacy and side-effect profile of brentuximab vedotin, she opted for the new treatment. After approval by the National Department of Health, the drug was imported via the named-patient-program (Millennium/Takeda). The treatment was commenced at the recommended dose (1.4 mg/m^2^) every 3 weeks. The neck tumor regressed dramatically with minimal toxicity. Follow-up PET/CT scans after 4 and 9 cycles of BV showed CR of the tumor (Figures 3(b) and 3(c)). The most recent PET/CT scanning confirmed continuous remission of the tumor for 11 months. The patient is in good health and receives close monitoring of remission status, with ASCT as an option for further salvage.

## 3. Discussion

Optimal management of RR-HL is a difficult clinical challenge with three conventional options of salvage treatment: radiotherapy, chemotherapy, and stem cell transplantation. 

For selected patients with confirmed local relapse, salvage radiotherapy is a very effective treatment modality with nearly 80% CR rate and 65% long-term local control in the GHSG report [5, 6]. But most patients will undergo second-line chemotherapy in order to obtain a second remission. Phase II trials of various salvage chemotherapy regimens had reported RR around 75%, with CR rate around 40%. However, there are no direct comparison of different regimens and no consensus on the standard second-line chemotherapy [7]. 

For patients with sensitive relapse, high-dose chemotherapy followed by ASCT is the current standard treatment based on two randomized phase III studies by the British National Lymphoma Investigation and the GHSG, reporting 3-year PFS around 50%. However, chemorefractory patients were underrepresented in these trials due to exclusion of most patients with rapid progression [8, 9].

Among the adverse prognostic factors identified in patients with RR-HL, remission duration after initial treatment was particularly important. In a report of salvage chemotherapy for RR-HL by the NCI Italy, primary refractory HL has the poorest eight-year survival of 8% (versus 28% for early relapse and 54% for late relapse) [10]. The five-year-survival rates of patients in the GHSG cohort were 26% for the primary refractory (progression during induction treatment or within 3 months after the end of treatment), 46% for the early relapse (between 3 and 12 months), and 71% for the late relapse (after 12 months) [11]. Patients achieved tumor remission after salvage chemotherapy then underwent ASCT which yielded a higher OS of 42% in the GHSG report. However, further exploratory landmark analysis suggested the favorable result of ASCT might be due to exclusion of poor-risked patients with refractory disease, poor-performance status, older age, insufficient stem cell harvest, or life-threatening toxicity on salvage treatment [11]. The GELA H89 study was designed to explore prospectively the strategy of early intensified chemotherapy followed by HDC/ASCT in patients of RR-HL and found the 5-year OS in primary refractory HL to be significantly lower than in other RR-HL patients (30% versus 74%, *P* = 0.0001) [4]. 

Considering the observed tradeoff between efficacy and toxicities of intensified chemotherapy and the suboptimal outcome of ASCT in refractory cases, more selective drug with higher therapeutic index is clearly needed for the poor-risked HL. Brentuximab vedotin (SGN 35) is a chimeric anti-CD30 antibody conjugated by a protease-cleavable linker to a microtubule-disrupting agent (monomethyl auristatin E; MMAE). After binding to cell-surface CD30, the MMAE is internalized, traffics to the lysosome, and then is released to disrupt microtubules, and to induce cell-cycle arrest and apoptosis. In the phase I dose escalation trial of 45 patients with relapsed/refractory CD30+ hematological malignancies, tumor regression was observed in 86% of cases [12]. The subsequent pivotal phase II trial (SG035-0003) in 102 patients of Hodgkin lymphoma with post-ASCT relapses achieved a 75% response rate (34% CR) and 96% disease control rate [13]. Considering the high proportions of poor risk features in these patients—primary refractory (in 72%), refractory to salvage therapy (in 42%), and early relapse after ASCT (in 71%)—the new drug seemed to be effective across all the high-risk groups. These favorable results led to accelerated approval by the US FDA in August 2011.

The safety profiles for BV are favorable compared with conventional cytotoxic chemotherapy. More common side effects are usually mild and include cough, fatigue, pyrexia, nausea, and peripheral neuropathy. The most clinical relevant toxicity is peripheral neuropathy, which is usually of sensory type, with an 80% spontaneous improvement rate. A rare but severe side effect of progressive multifocal leukoencephalopathy was reported recently in 3 cases (among 2000+ patients). Clinician should stay alert to and stop the medication with any newly onset CNS signs and symptoms.

Exciting reports about brentuximab vedotin are continuously emerging. In patients with post-ASCT relapse, a recent study confirmed the superior survival with BV than any other salvage treatments (median: 91.49 mo versus 27.99 mo, *P* = 0.0004). In this report, patients achieving CR after BV had a prolonged progression-free survival of 29 months [14]. Other exciting reports included higher CR rate of BV (34%) than ASCT (15%) in RR-HL [15], a role in facilitation of reduced-intensity allotransplantation [16] and effective salvage of relapse after allo-SCT [17].

Many researches are currently ongoing to explore the expanding roles of this new drug in management of HL and other CD30+ malignancies. However, the role of BV in salvage treatment of primary refractory HL is an under-explored area which needs further clinical studies. This case scenario shows good efficacy of BV in primary refractory HL and suggests the potential use in this poor-risk group. Hopefully, with optimal incorporation of BV into current treatment modalities, the survival of primary refractory and other poor-risk Hodgkin lymphoma will be further improved. 

## Abbreviations

BV: Brentuximab vedotin
RR-HL: Relapsed or refractory Hodgkin lymphoma
GHSG: German Hodgkin Study Group
PFS: Progression-free survival
OS: Overall survival
ASCT: Autologous stem cell transplantation
CR: Complete remission.
